# Supplementary material for: Retinoic acid catabolizing enzyme CYP26C1 is a genetic modifier in SHOX deficiency
Source: EMBO Mol Med. 2016 Nov 14;8(12):1455–69. doi: 10.15252/emmm.201606623 (PMC5167135; doi:10.15252/emmm.201606623)
Supplement: Supplementary file 1 — Appendix [file EMMM-8-1455-s001.pdf]

Appendix Figures

Retinoic acid catabolizing enzyme CYP26C1 is a genetic modifier in SHOX deficiency

Antonino Montalbano[1], Lonny Juergensen[2], Ralph Roeth[1], Birgit Weiss[1],Maki Fukami[3],Susanne Fricke-Otto[4],Gerhard Binder[5],Tsutomu Ogata[6], Eva Decker[7],Gudrun Nuernberg[8,9], David Hassel[2], Gudrun A. Rappold[1,10*].

1. Department of Human Molecular Genetics, Heidelberg University, Heidelberg, 69120, Germany.
2. Department of Internal Medicine III - Cardiology, Heidelberg University Hospital, Heidelberg, 69120, Germany.
3. Department of Molecular Endocrinology, National Research Institute for Child Health and Development, Tokyo, 157-8535, Japan.
4. Children's Hospital Krefeld, Krefeld, 47805, Germany.
5. Children's Hospital, University of Tübingen, Tübingen, 72076, Germany.
6. Department of Pediatrics, Hamamatsu University School of Medicine, Hamamatsu, 431-3192, Japan.
7. Bioscientia Center for Human Genetics, Ingelheim, 55218, Germany.
8. Center for Molecular Medicine, Cologne, 50931, Germany.
9. Cologne Center for Genomics, Cologne, 50931, Germany.
10. Interdisciplinary Centre for Neurosciences (IZN), University of Heidelberg, Heidelberg, 69120, Germany.

Content:

Appendix Figures S1-S8.. **2-9**

Appendix Figures legends.. **10**

Appendix Figure S1

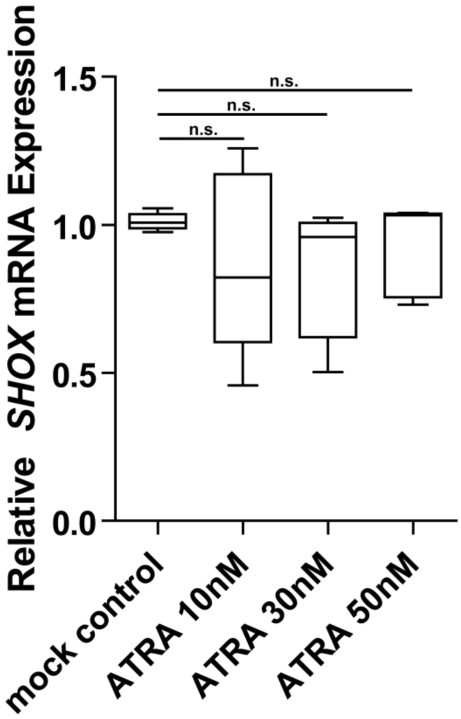

Appendix Figure S2

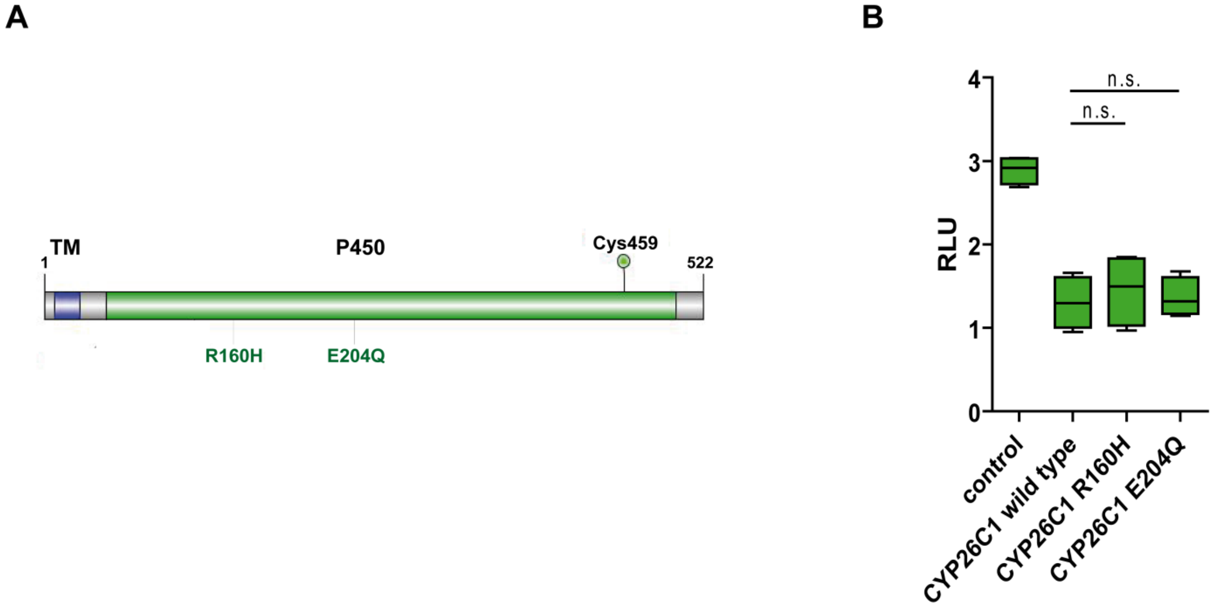

Appendix Figure S3

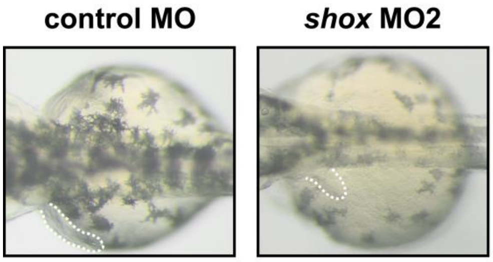

Appendix Figure S4

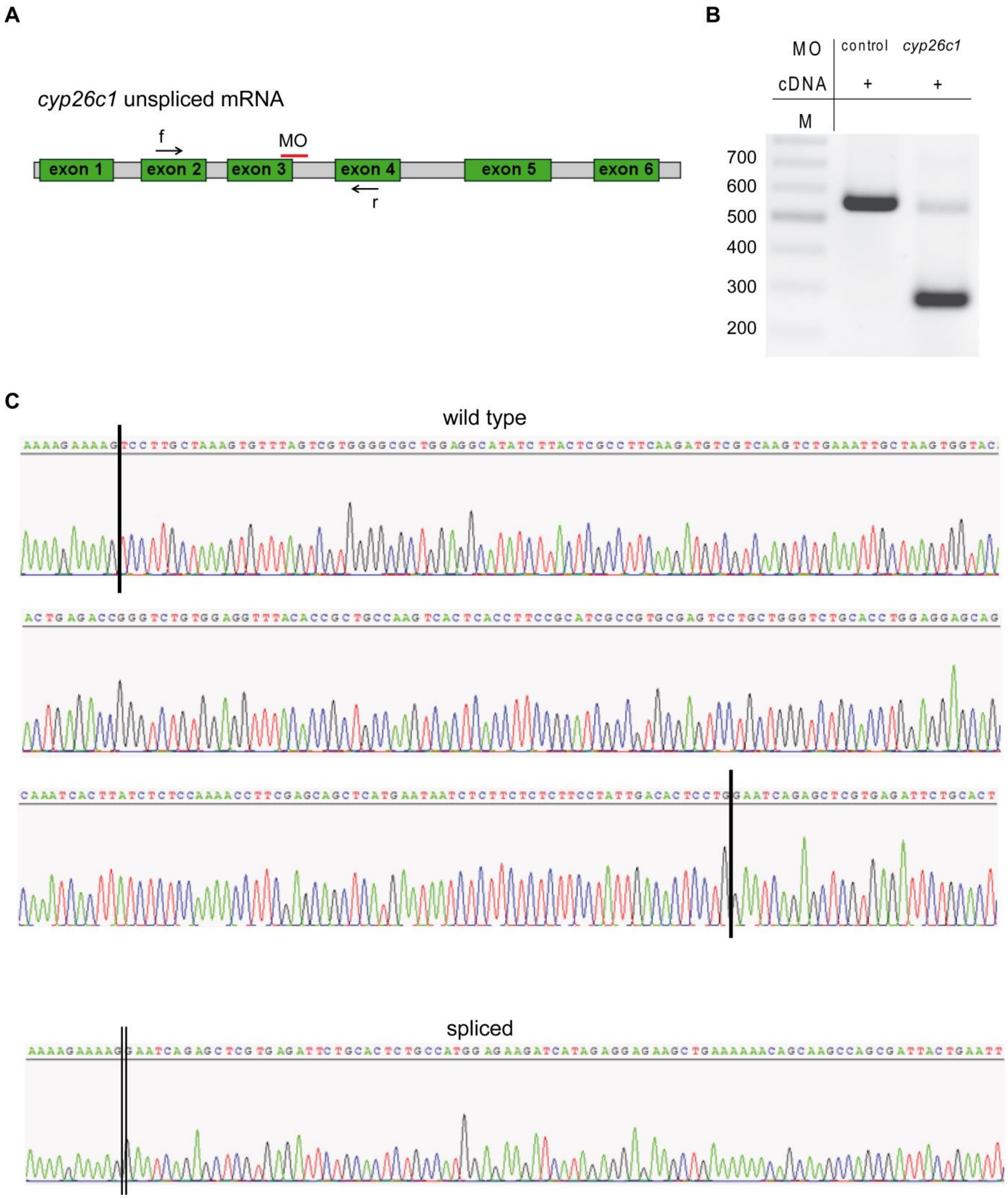

Appendix Figure S5

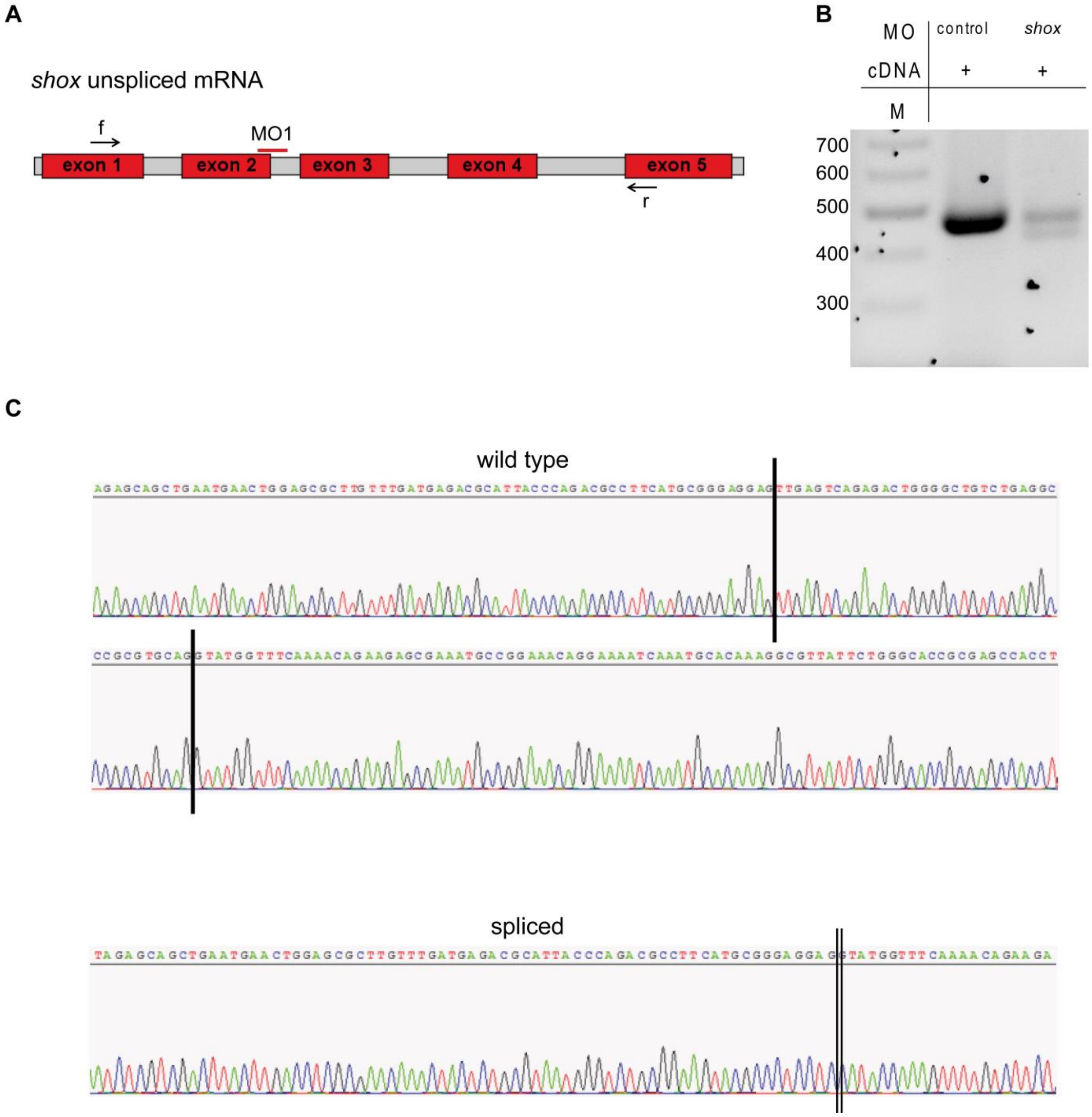

Appendix Figure S6

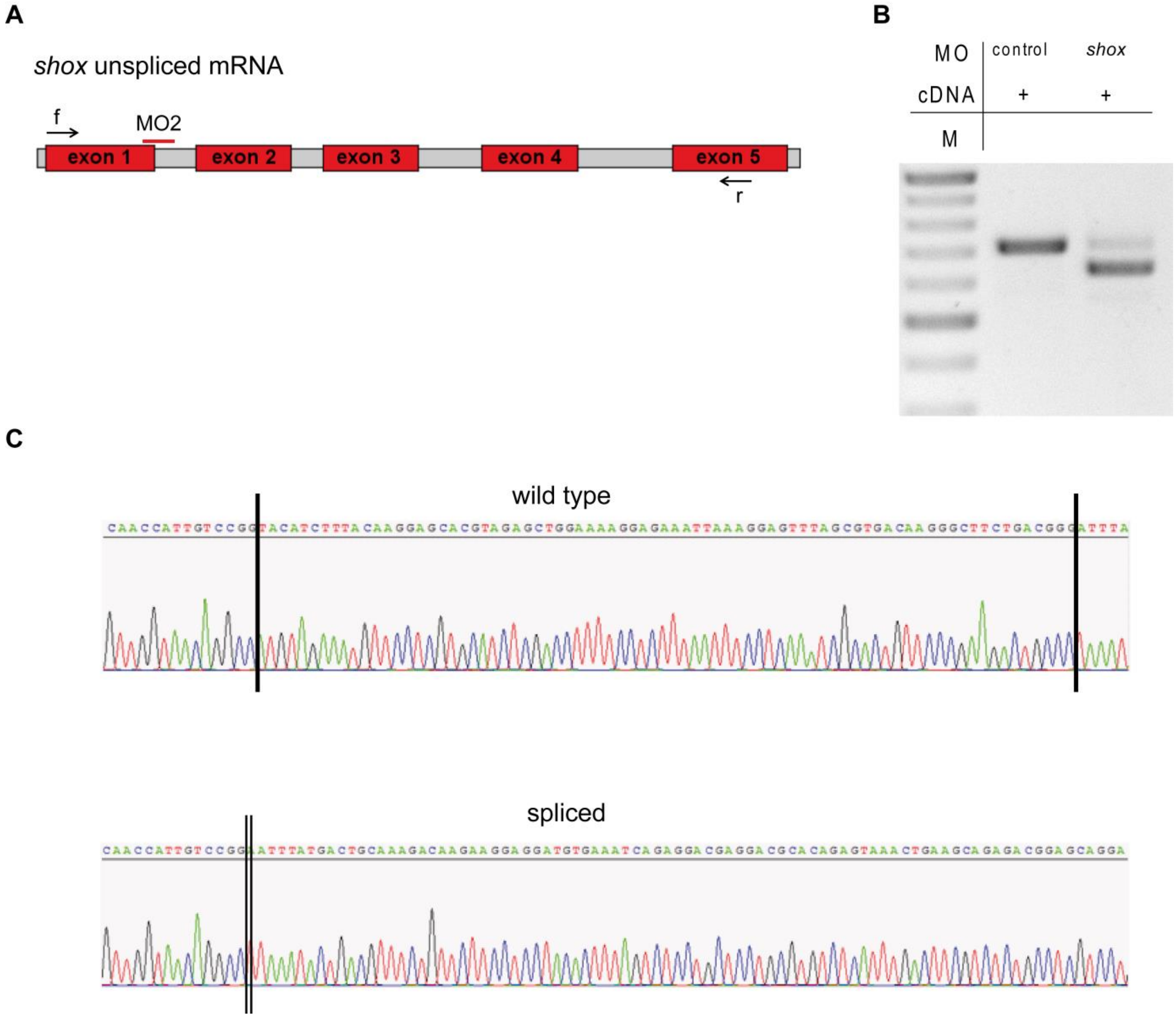

Appendix Figure S7

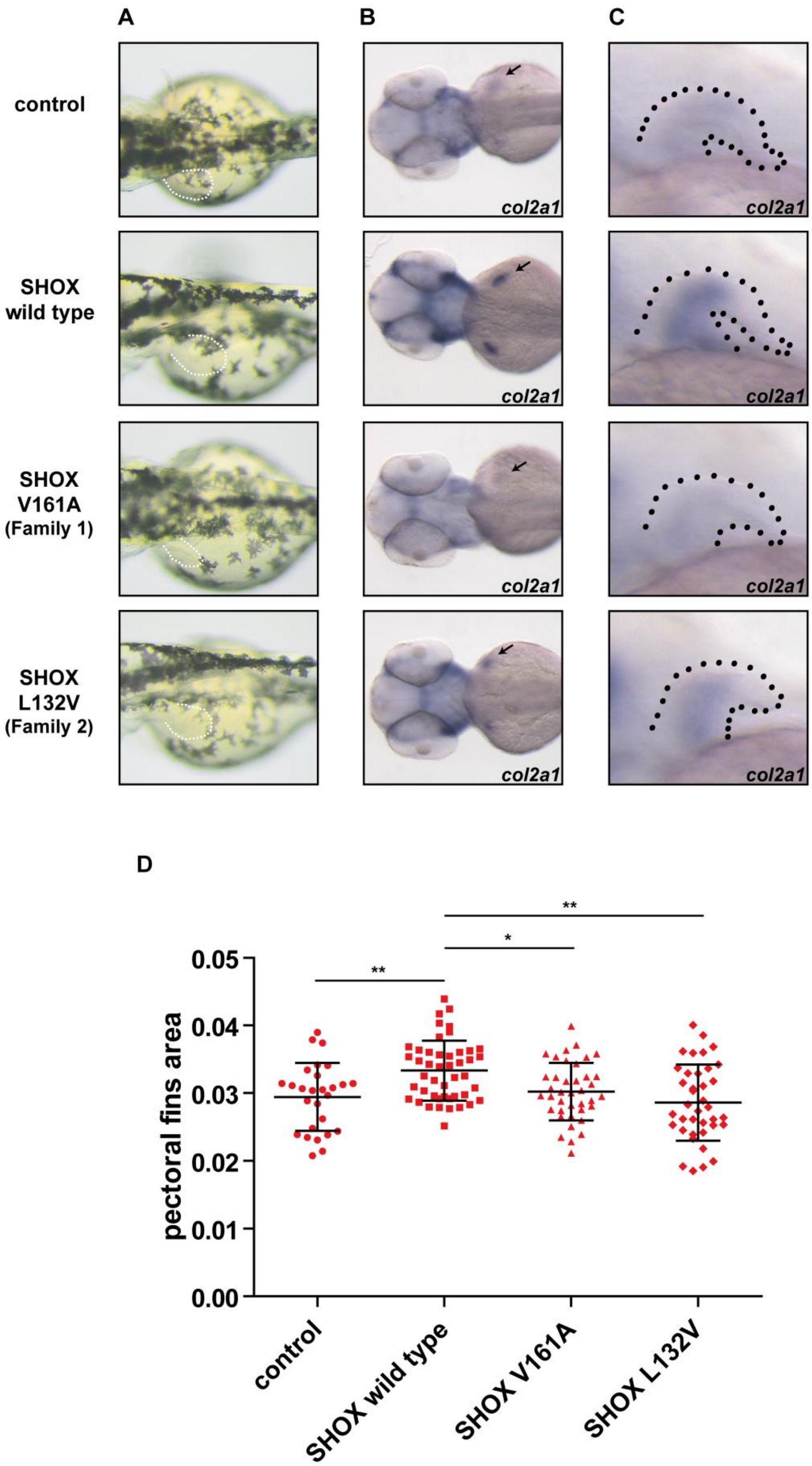

Appendix Figure S8

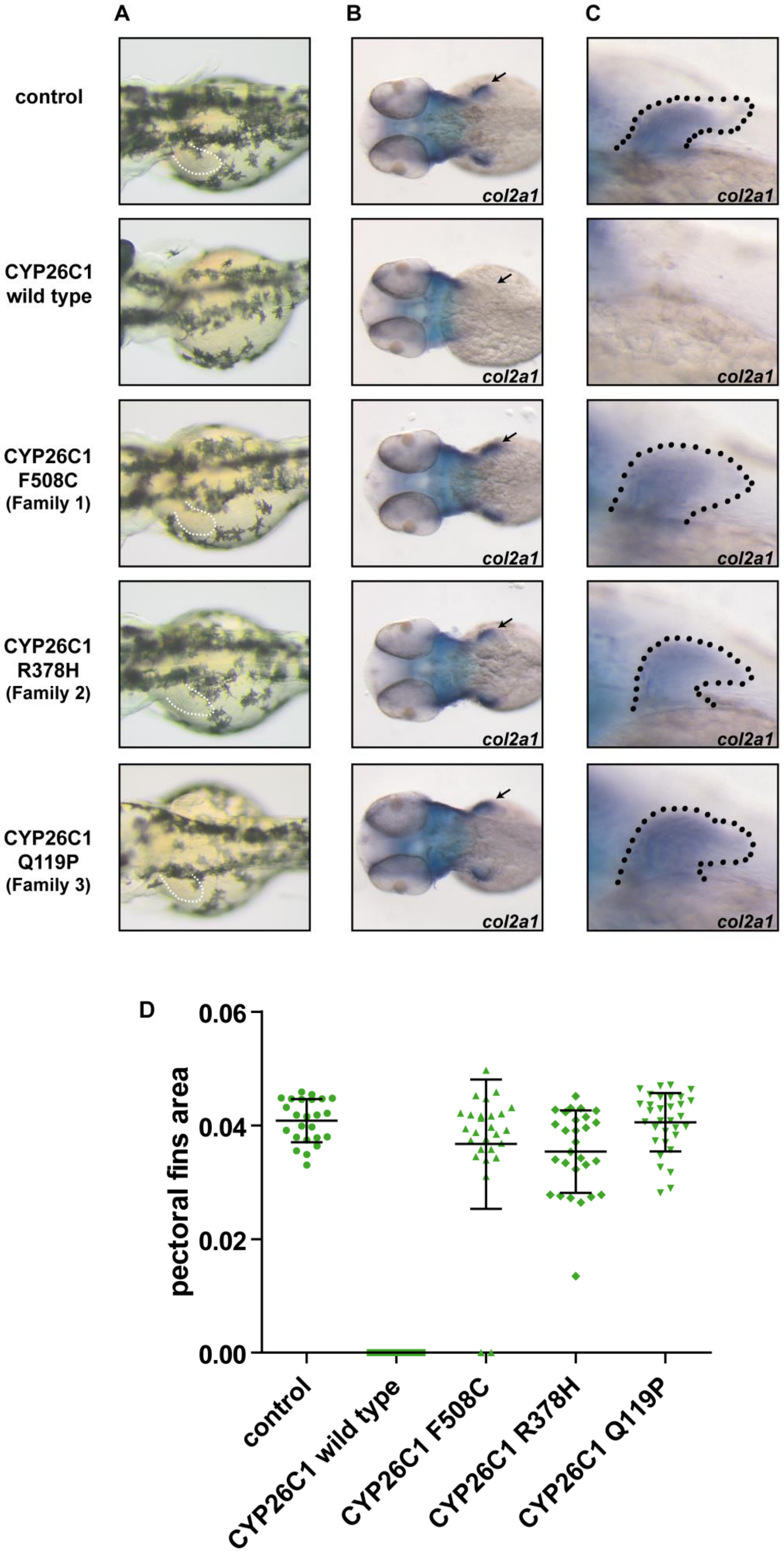

Appendix Figures Legends

Figure S1 – Treatment of human primary chondrocytes with RA concentrations within the physiological levels.

Relative expression of *SHOX* mRNA normalized to the reference genes *SDHA* and *HPRT* in human primary chondrocytes treated with ATRA 10, 30, and 50 nM for 6h (n = 5).

Data information: The box represents the interquartile range. The whiskers represent Min to Max. n.s., not significant, two-tailed Mann-Whitney non-parametric *t* test.

Figure S2 - CYP26C1 protein representation and luciferase assay of the variants found in normal height controls.

Variants found in CYP26C1 in control normal height individuals are indicated in green. Cignal-RARE system luciferase assays to test the impact of CYP26C1 variants on its RA degradation activity were performed in U2OS cells treated with 250 nM all-trans retinoic acid (ATRA) for 24 hours (n = 4). Experiments were performed in triplicates. pIRES2-EGFP empty vector was used as control. The residue C459 represents the Iron binding residue (Q6VOL0, UniProtKB). TM, Transmembrane helix; P450, cytochrome p450 domain. RLU, Relative Light Units.

Data information: The box represents the interquartile range. The whiskers represent Min to Max. n.s., not significant, two-tailed Mann-Whitney non-parametric *t* test.

Figure S3 - Pattern of defects in zebrafish embryos injected with anti-*shox* MO2.

Wild type embryos injected with control MO or with *shox* MO2 (n = 30 embryos). Dorsal view of the embryos at 55 hpf. Dotted line, pectoral fins.

Figure S4 - *cyp26c1* MO efficacy analysis.

A. Scheme of *cyp26c1* unspliced mRNA. Red bar, region targeted by *cyp26c1* MO; arrows, position of the primers used to analyze MO efficacy; f, forward primer; r, reverse primer.

B. RT-PCR analysis of *cyp26c1* MO efficacy. The 530 bp band represents the expected wild type product. Samples were obtained from 24 hpf embryos.

C. RT-PCR products were cloned in pSTBlue-1 and sequenced. *cyp26c1* MO leads to the exclusion of exon 3. Black vertical segments enclose the out-spliced sequence.

Figure S5 –*shox* MO1 efficacy analysis.

A. Scheme of *shox* unspliced mRNA. Red bar, region targeted by *shox* MO1; arrows, position of the primers used to analyze MO efficacy; f, forward primer; r, reverse primer.

B. RT-PCR analysis of *shox* MO1 efficacy. The 430 bp band represents the expected wild type product. Samples were obtained from 24 hpf embryos.

C. RT-PCR products were cloned in pSTBlue-1 and sequenced. *shox* MO1 leads to a spliced product leading to frameshift, p.L142Rfs37*. Black vertical segments enclose the out-spliced sequence.

Figure S6 –*shox* MO2 efficacy analysis.

A. Scheme of *shox* unspliced mRNA. Red bar, region targeted by *shox* MO2; arrows, position of the primers used to analyze MO efficacy; f, forward primer; r, reverse primer.

B. RT-PCR analysis of *shox* MO2 efficacy. The 680 bp band represents the expected wild type product. Samples were obtained from 24 hpf embryos.

C. RT-PCR products were cloned in pSTBlue-1 and sequenced. MO2 leads to a spliced product leading to frameshift, p.V59Pfs11*. Black vertical segments enclose the out-spliced sequence.

Figure S7 – Functional significance of the *SHOX* variants identified in families 1 and 2 in zebrafish embryos.

A-C. Wild type embryos injected with sense-capped RNA coding for human SHOX wild type or the variants identified in families 1 and 2, V161A (family 1) and L132V (family 2). Note that the patient in family 3 presented a *SHOX* deletion. (A) Dorsal views of the embryos at 55 hours post fertilization (hpf). Embryos injected with *SHOX* wild type RNA displayed longer pectoral fins when compared to control and SHOX variants V161A and L132V (n = 27-46 per condition). These results are in agreement with a preview study reporting that overexpression of *Shox* in chicken wings increases the length of skeletal elements (Tiecke et al, 2006). Dotted line, pectoral fins. (B, C) Dorsal view and magnification on the pectoral fins of *col2a1* expression at 55 hpf. Arrow and dotted line indicate the pectoral fin. Embryos injected with *SHOX* wild type RNA displayed increased *col2a1* expression.

D. Pectoral fins area was measured by imageJ (n = 27-46 embryos per condition).

Data information: Data are shown as means ± SD. SHOX wild type vs SHOX V161A, * p-value = 0.019. SHOX wild type vs control, ** p-value = 0.006. SHOX wild type vs SHOX L132V, ** p-value = 0.008. One-way ANOVA Bonferroni's multiple comparison test.

Figure S8 – Functional significance of the *CYP26C1* variants identified in families 1-3 in zebrafish embryos.

A-C. Wild type embryos injected with sense-capped RNA coding for human CYP26C1 wild type or the variants identified in families 1-3, F508C (family 1), R378H (family 2), Q119P (family 3). (A) Dorsal views of the embryos at 55 hours post fertilization (hpf). Embryos injected with *CYP26C1* wild type RNA displayed absent pectoral fins. Ectopic overexpression of CYP26C1 may lead to RA deficiency. Deficiency of RA at early stages of pectoral fin development has been previously shown to lead to similar phenotypes (Begemann et al, 2001). As shown in Fig 2, the *CYP26C1* variants identified in families 1-3 disrupt CYP26C1 RA degradation activity and overexpression, therefore, does not alter RA levels within the embryos resulting in normal pectoral fins development (n = 24-32 per condition). Dotted line, pectoral fins. (B, C) Dorsal view and magnification on the pectoral fins of *col2a1* expression at 55 hpf. Arrow and dotted line indicate the pectoral fin.

D. Pectoral fins area was measured by imageJ (n = 24-32 embryos per condition).
