# Supplementary material for: Retinoic acid catabolizing enzyme CYP26C1 is a genetic modifier in SHOX deficiency
Source: EMBO Mol Med. 2016 Nov 14;8(12):1455–69. doi: 10.15252/emmm.201606623 (PMC5167135; doi:10.15252/emmm.201606623)
Supplement: Supplementary file 2 — Expanded View Figures PDF [file EMMM-8-1455-s002.pdf]

Expanded View Figures

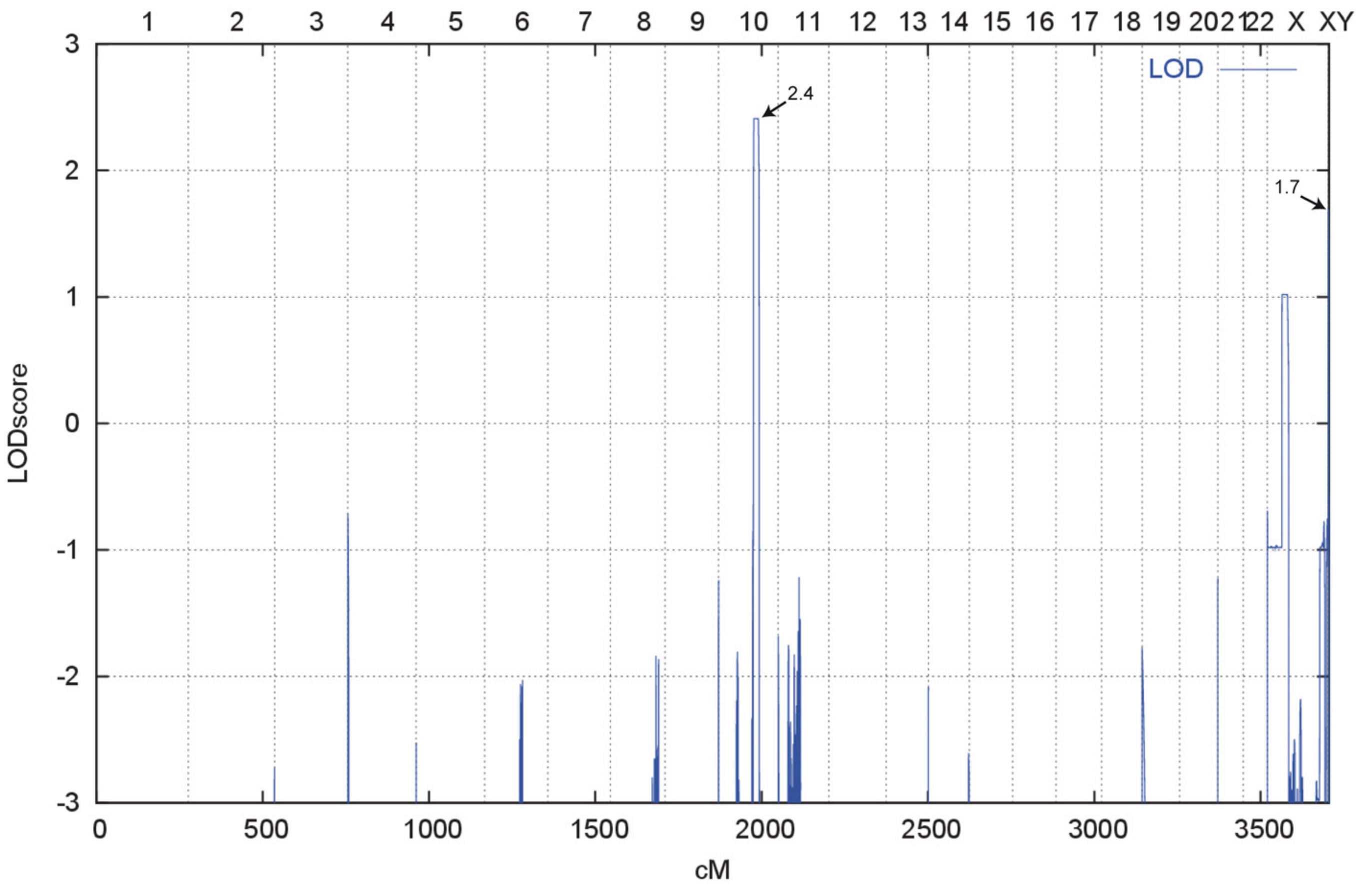

Figure EV1. Schematic representation of genome-wide LOD score calculations.
LOD scores calculated with ALLEGRO are given along the *y*-axis relative to genomic position cM (centiMorgan) on the *x*-axis. Note the highest peak (LOD score 2.4) in the region on chromosome 10 and a second lower peak in the XY PAR1 (LOD score 1.7).

© 2016 The Authors

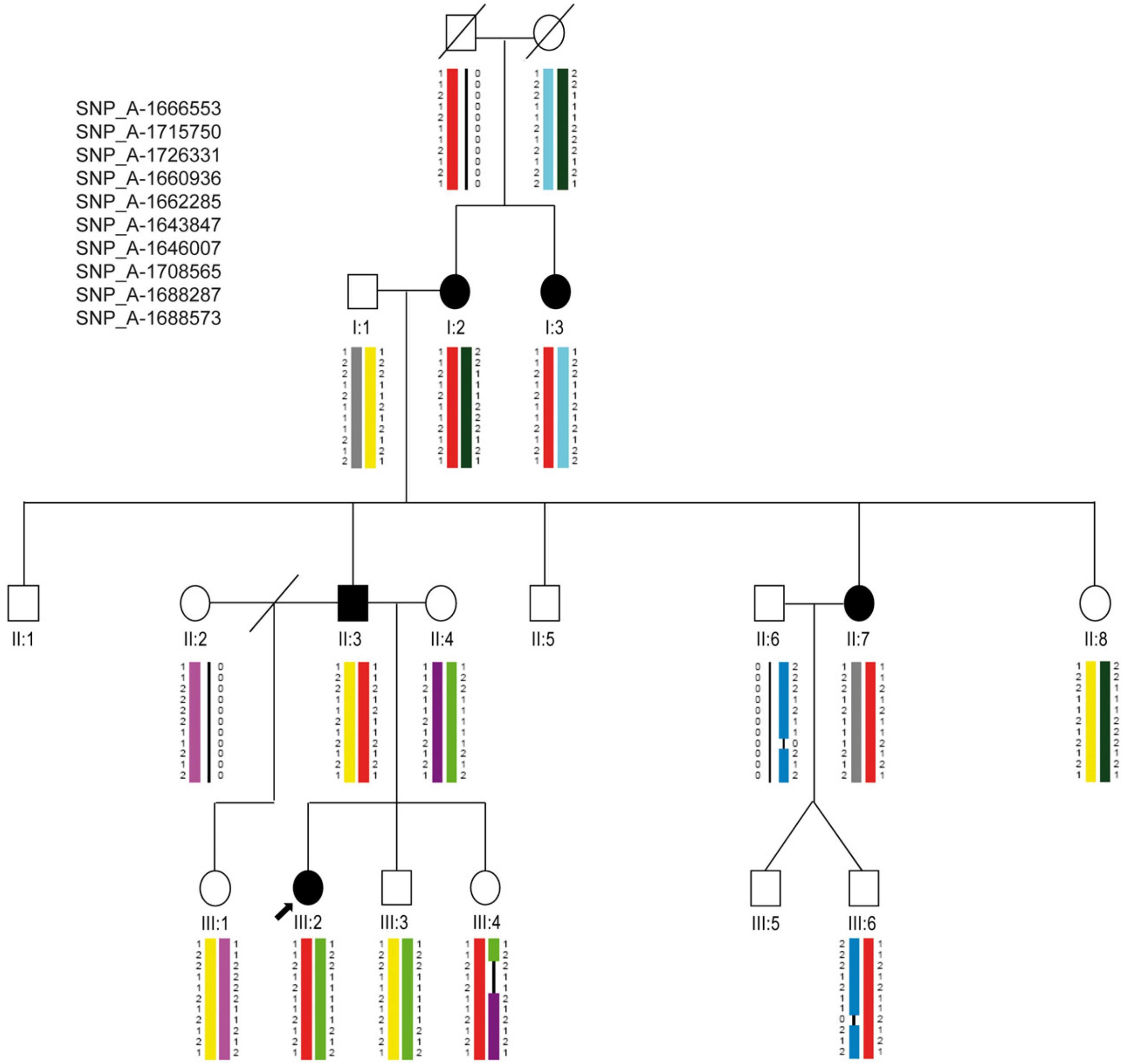

Figure EV2. Haplotype reconstruction for the PAR1 region.
Pedigree of the family with associated SNPs in the pseudoautosomal region 1 (PAR1) of chromosome X is represented. A total LOD score of 1.7 was identified between the flanking markers rs3995646 and rs5939344 and covered a 2.02 Mb sequence (chrX:706800-2735491; hg19). Filled symbol, LWD-affected individual; symbol with a slash, deceased individual; slash, divorced; arrow, index patient. Colored chromosomal regions show traceable inheritance: red color regions, common haplotype co-segregating with LWD; black lines, regions affected by a crossing over of unknown location.

Figure EV3. Haplotype reconstruction for chromosome 10.
Pedigree of the family with associated SNPs co-segregating with the disease phenotype on chromosome 10 is represented. A total LOD score of 2.4 was identified between the flanking markers rs10509480 and rs10509758 and covered a 19.2 Mb region (chr10:85477515-104681710; hg19). Filled symbol, LWD-affected individual; symbol with a slash, deceased individual; slash, divorced; arrow, index patient. Colored chromosomal regions show traceable inheritance; red color regions, common haplotype co-segregating with LWD; black lines, regions affected by a crossing over of unknown location.

© 2016 The Authors

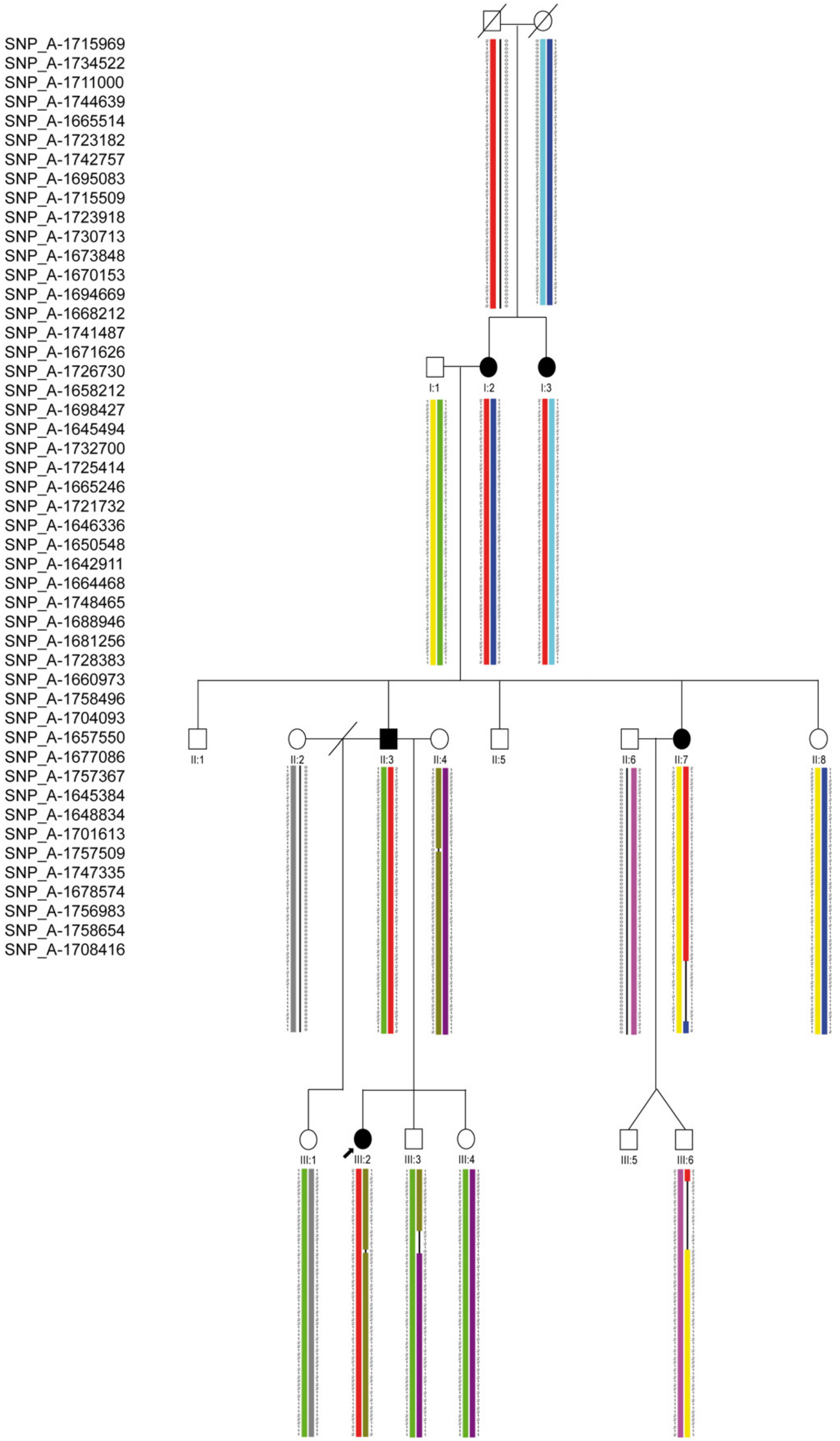

Figure EV3.

 © 2016 The Authors
