# Supplementary material for: Retinoic acid catabolizing enzyme CYP26C1 is a genetic modifier in SHOX deficiency
Source: EMBO Mol Med. 2016 Nov 14;8(12):1455–69. doi: 10.15252/emmm.201606623 (PMC5167135; doi:10.15252/emmm.201606623)
Supplement: Supplementary file 3 — Table EV1 [file EMMM-8-1455-s003.docx]

| **Table EV1. Families’ 1-3 phenotypic data.**  Data information: *, index patient; M, male; F, female; SD, standard deviation; LA, lower arm; N.A., not available; **, this measure refers to the index patient when she was 14 years old. Madelung deformity: +, present; -, not present; (+), borderline. *SHOX* or *CYP26C1* locus: +, wild type; N.A., not available. | | | | | | | | |
| --- | --- | --- | --- | --- | --- | --- | --- | --- |
|  |  |  |  |  |  |  |  |  |
|  |  |  |  |  |  |  |  |  |
| **Family** | **Patient ID** | **Age** | **Gender** | **Height SD** | **LA SD** | **Madelung deformity** | ***SHOX*** | ***CYP26C1*** |
|  |  |  |  |  |  |  |  |  |
| **Family 1** | I:1 | 72 | M | -1.85 | +0.57 | - | +/+ | +/+ |
|  | I:2 | 72 | F | -4.51 | -2.21 | + | p.V161A/+ | p.F508C/+ |
|  | I:3 | 81 | F | -3.73 | N.A. | + | p.V161A/+ | p.F508C/+ |
|  | II:1 | 51 | M | -1.23 | N.A. | - | N.A. | N.A. |
|  | II:2 | N.A. | F | N.A. | N.A. | - | N.A. | N.A. |
|  | II:3 | 46 | M | -2.63 | -2.00 | (+) | p.V161A/+ | p.F508C/+ |
|  | II:4 | 42 | F | -3.33 | -1.15 | - | +/+ | +/+ |
|  | II:5 | 40 | M | -0.92 | N.A. | - | N.A. | N.A. |
|  | II:6 | N.A. | M | N.A. | N.A. | - | N.A. | N.A. |
|  | II:7 | 37 | F | -3.14 | N.A. | + | p.V161A/+ | p.F508C/+ |
|  | II:8 | 32 | F | -2.75 | -0.50 | - | +/+ | +/+ |
|  | III:1 | 21 | F | -3.73 | -1.20 | - | +/+ | +/+ |
|  | III:2* | 8.9 | F | -3.60 | -2.72** | + | p.V161A/+ | p.F508C/+ |
|  | III:3 | 12.9 | M | -0.62 | +0.10 | - | +/+ | +/+ |
|  | III:4 | 8.5 | F | -1.96 | -1.67 | - | p.V161A/+ | +/+ |
|  | III:5 | 16 | M | -0.43 | N.A. | - | p.V161A/+ | +/+ |
|  | III:6 | 16 | M | -0.72 | N.A. | - | p.V161A/+ | +/+ |
|  |  |  |  |  |  |  |  |  |
| **Family 2** | I:1 | N.A. | M | N.A. | N.A. | - | L132V/+ | +/+ |
|  | I:2 | N.A. | F | N.A. | N.A. | - | +/+ | N.A. |
|  | II:1 | N.A. | F | N.A. | N.A. | - | L132V/+ | +/+ |
|  | II:2 | 6 | F | -3.38 | -4.71 | + | L132V/+ | R378H/+ |
|  |  |  |  |  |  |  |  |  |
| **Family 3** | I:1 | 51 | M | +0.1 | N.A. | - | +/+ | Q119P/+ |
|  | I:2 | 47 | F | -0.2 | +0.8 | - | +/+ | +/+ |
|  | II:1 | 26 | M | -0.1 | +0.5 | - | +/+ | +/+ |
|  | II:2 | 16 | F | -2.3 | -3,4 | + | del/+ | Q119P/+ |
